# Medical College Notes

**Published:** 1889-12

**Authors:** 


### Medical College Notes.

AT A recent meeting of the Faculty of Niagara University Medical College, Dr. William C. Krauss was elected Lecturer on Pathology. Dr. Krauss is well fitted for the work assigned him, having recently returned from the laboratories of the Old World, where he enjoyed the teachings of men like Virchow, Bollinger, Mendel, Gombault, etc. His contributions to Pathology, and more especially to Neuropathology, during the past few years, show him to be an earnest and enthusiastic worker. Dr. Krauss limits his practice in this city to the diseases of the nervous system.

---

MR. HENRY K. JESSEL, assistant to Professor Pitt, in the Department of Natural Sciences, at the Buffalo High School, has been elected Assistant in General Chemistry and Physics in the Medical Department of Niagara University. Mr. Jessel is a graduate of Cornell University in the class of 1889, and brings to his new duties the earnestness and energy of youth, combined with fine scholarship and an aptitude for teaching, which gives assurance of success in his new field of labor.

